# Characterization of ligand-induced thermal stability of the human organic cation transporter 2 (OCT2)

**DOI:** 10.3389/fphar.2023.1154213

**Published:** 2023-03-16

**Authors:** Max Maane, Fangrui Xiu, Peter Bellstedt, Gerd A. Kullak-Ublick, Michele Visentin

**Affiliations:** ^1^ Department of Clinical Pharmacology and Toxicology, University Hospital Zurich, University of Zurich, Zurich, Switzerland; ^2^ Affiliated Hospital of Shandong University of Traditional Chinese Medicine, Shandong University of Traditional Chinese Medicine, Jinan, China; ^3^ Institute of Clinical Chemistry, University Hospital Zurich, University of Zurich, Zurich, Switzerland

**Keywords:** *cis*-inhibition, OCT, organic cation transporter, SLC, thermal shift assay, thermodynamic stabilization, TSA

## Abstract

**Introduction:** The human organic cation transporter 2 (OCT2) is involved in the transport of endogenous quaternary amines and positively charged drugs across the basolateral membrane of proximal tubular cells. In the absence of a structure, the progress in unraveling the molecular basis of OCT2 substrate specificity is hampered by the unique complexity of OCT2 binding pocket, which seemingly contains multiple allosteric binding sites for different substrates. Here, we used the thermal shift assay (TSA) to better understand the thermodynamics governing OCT2 binding to different ligands.

**Methods:** Molecular modelling and *in silico* docking of different ligands revealed two distinct binding sites at OCT2 outer part of the cleft. The predicted interactions were assessed by *cis*-inhibition assay using [^3^H]1-methyl-4-phenylpyridinium ([^3^H]MPP^+^) as a model substrate, or by measuring the uptake of radiolabeled ligands in intact cells. Crude membranes from HEK293 cells harboring human OCT2 (OCT2-HEK293) were solubilized in n-Dodecyl-β-D-Maltopyranoside (DDM), incubated with the ligand, heated over a temperature gradient, and then pelleted to remove heat-induced aggregates. The OCT2 in the supernatant was detected by western blot.

**Results:** Among the compounds tested, *cis*-inhibition and TSA assays showed partly overlapping results. Gentamicin and methotrexate (MTX) did not inhibit [^3^H]MPP^+^ uptake but significantly increased the thermal stabilization of OCT2. Conversely, amiloride completely inhibited [^3^H]MPP^+^ uptake but did not affect OCT2 thermal stabilization. [^3^H]MTX intracellular level was significantly higher in OCT2-HEK293 cells than in wild type cells. The magnitude of the thermal shift (ΔT_m_) did not provide information on the binding. Ligands with similar affinity showed markedly different ΔT_m_, indicating different enthalpic and entropic contributions for similar binding affinities. The ΔT_m_ positively correlated with ligand molecular weight/chemical complexity, which typically has high entropic costs, suggesting that large ΔT_m_ reflect a larger displacement of bound water molecules.

**Discussion:** In conclusion, TSA might represent a viable approach to expand our knowledge on OCT2 binding descriptors.

## 1 Introduction

The human organic cation transporter 2 (OCT2) belonging to the SLC superfamily of solute carriers, is the main membrane transporter of organic cations in the kidney, where it localizes to the basolateral membrane of proximal tubular cells of S1 and S2 segments (Gorboulev et al., 1997; Karbach et al., 2000; Motohashi et al., 2002; Visentin et al., 2018). Preferred, but not the sole substrates of OCT2 are positively charged compounds. The list includes nutrients such as thiamin (vitamin B1), choline, and L-carnitine, breakdown products such as creatinine and trimethylamine oxide (TMAO), and the neurotransmitters serotonin and norepinephrine (Koepsell, 2013; Gai et al., 2016; Visentin et al., 2017; Visentin et al., 2018). OCT2 is also considered to mediate the entry step of the tubular secretion of many widely prescribed drugs, to the point that regulatory authorities included OCT2 in the panel of clinically relevant transporters, which must be screened for *in vitro* prediction of drug-drug interactions (DDIs) in preclinical development (EMA, 2012; FDA, 2017).

Extensive data from patch-clamp and trans-stimulation experiments focused on rat Oct1 and Oct2 gathered over the years indicate that OCT-mediated transport is driven by the membrane potential and the substrate electrochemical gradient across the plasma membrane. Simply put, in cells with normal inside-negative membrane potential cation uptake is energetically preferred whereas cation efflux can only occur in depolarized cells or when the intracellular concentration of the substrate far exceeds the extracellular one, overcoming the inside-negative membrane potential (Koepsell, 2019). OCT transport follows the alternating-access mechanism typical of secondary transporters, in which substrates are carried across the membrane as the protein cycles between inward- and outward-facing conformations (Forrest et al., 2011; Koepsell, 2019). Conversely, OCT ligand recognition is somewhat peculiar, whereby structurally different compounds can bind simultaneously and influence each other’s binding affinity, suggesting the presence of multiple allosteric binding sites sufficiently spaced apart to allow non-exclusive binding (Harper and Wright, 2013; Keller et al., 2019; Hormann et al., 2020; Sutter et al., 2021). Such a model has been proposed mainly based on *cis*-inhibition studies and direct measurements of affinity constants (K_m_), two invaluable approaches for ligand screening and biochemical characterization of the transport mechanism but that provide little information on the molecular mechanisms of the binding. Moreover, the *cis*-inhibition-based approach exclusively describes the ligand interaction with the transporter in the substrate-bound state, which might not be informative of the ligand interaction to the unbound transporter, especially for transporters like OCT2 that are subjected to substrate-induced allosteric modifications. Finally, K_m_ values are descriptive constants of transport rather than binding, hence the net result of substrate binding and dissociation, and time constants of conformational changes during the transport cycle, hence not necessarily the sole reflection of binding (Koepsell, 2015; Koepsell, 2021).

In the absence of cryo-EM structures of OCT2, which would allow the definition of the ligand binding pocket and ligand-dependent conformational changes, a direct approach to study binding, rather than transport exploits the principle of thermodynamic stabilization that a ligand confers to its target protein upon binding (Matulis et al., 2005; Niesen et al., 2007). Thermal shift assay (TSA) has been extensively used to monitor ligand engagement to purified soluble proteins, but less frequently applied to the study of transmembrane proteins, mainly because they are often difficult to produce and isolate (Lo et al., 2004). More recently, it has been shown that TSA can be applied to monitor ligand engagement to both soluble and transmembrane proteins in unpurified samples by using cellular systems overexpressing the GFP-fusion target or by coupling the TSA to quantitative Western blotting (Martinez Molina et al., 2013; Jafari et al., 2014; Chatzikyriakidou et al., 2021). Here we explored the suitability of TSA to study the binding to OCT2 to different ligands and the impact of different chemical properties (e.g., hydrophobicity) on OCT2 thermodynamic stabilization.

## 2 Materials and methods

### 2.1 Reagents

(^3^H) N-methyl-4-phenylpyridinium acetate (RCTT0970, specific activity: 80.4 Ci/mmol) was purchased from RC Tritec (Teufen, Switzerland). Methotrexate [3',5',7-^3^H(N)] disodium salt (MR701, specific activity: 31.8 Ci/mmol) was acquired from Moravek Biochemicals (Brea, CA, United States). 1-Methyl-4-phenylpyridinium iodide (D048, MPP^+^), methotrexate hydrate (M8407, MTX), metformin hydrochloride (PHR1084), cimetidine (C4522), gentamicin sulfate (G3632), pravastatin sodium (P4498), amiloride hydrochloride hydrate (A7410) were purchased from Sigma-Aldrich (St. Louis, MO, United States). Dolutegravir sodium (CS-3496) was bought from Chemscene (Monmouth Junction, NJ, United States). Poly-D-Lysine was obtained from Corning (Bedford, MA, United States).

### 2.2 Cell culture

Wild-type human embryonic kidney cortex 293 cells (WT-HEK293) (CRL-1573, American Type Culture Collection, Rockville, MD, United States) and HEK293 cells that were stably transfected with the coding sequence of human OCT2 (OCT2-HEK293) were cultured in Dulbecco’s Modified Eagle Medium (DMEM) (ThermoFisher Scientific, Waltham, MA, United States). The cells were cultured at 37°C in a humidified atmosphere of 5% CO_2_. The medium contained 100 U/mL penicillin and 100 µg/mL streptomycin (ThermoFisher Scientific, Waltham, MA, United States) and 10% fetal bovine serum (Biowest, Nuaillé, France). The OCT2-HEK293 cells were grown under selective pressure with Geneticin G418 (ThermoFisher Scientific, Waltham, MA, United States) at the extracellular concentration of 400 µg/mL.

### 2.3 Transport assay

Cells were seeded onto 3.5-cm dishes that were previously coated with 0.1 mg/mL poly-D-Lysine at the density of 0.5 × 10^6^ cells/dish. After 72 h, cells were washed with transport buffer (5.3 mM KCl, 1 mM NaH_2_PO_4_, 0.8 mM MgSO_4_, 5.5 mM D-Glucose, 20 mM HEPES, 116.4 mM NaCl, adjusted to pH 7.4 with Tris base) pre-warmed at 37°C. Then, 0.5 mL of pre-warmed transport buffer in which non-radiolabeled and radiolabeled substrate were mixed to achieve a specific activity of 250-500 cpm/pmol, were added to the dish. The uptake was terminated after 10 s by multiple washing with ice-cold transport buffer. Cells were lysed in 1 mL of 1% (w/v) Triton X-100 and then 0.5 mL of the lysate was mixed with 3 mL of scintillation liquid Ultima Gold™ (Perkin Elmer, Waltham, MA, United States) and radioactivity was measured in a β-counter (Tri-Carb 2250 CA, Canberra Packard, Schwadorf, Austria). Twenty-5 µL of the lysate were used for the bicinchoninic acid protein assay (Interchim, Montluçon Cedex, France) and absorbance at 560 nm was measured with the GloMax Multi Detection System (Promega, Madison, WI, United States).

### 2.4 Crude membrane preparation

OCT2-HEK293 were seeded onto eight 10-cm dishes pre-coated with 0.1 mg/mL poly-D-Lysine. Each nearly confluent dish was rinsed three times with 10 mL ice-cold 0.9% (w/v) NaCl solution, and once with 10 mL ice-cold 250 mM sucrose. Then, cells were gently scraped in 3 mL of ice-cold 5 mM sucrose containing a cocktail of protease inhibitors (Complete Mini—Roche Diagnostics GmbH, Mannheim, Germany). The cell suspensions obtained from each plate were pooled into a pre-chilled tight-fitting glass-teflon potter for mechanical homogenization. The homogenate was subsequently centrifuged at 900 g_av_ for 10 min at 4°C to pellet unbroken cells and nuclei. This was followed by another step of centrifugation of the supernatant at 8,500 g_av_ for 20 min at 4°C to remove large organelles (e.g., mitochondria). The supernatant was then centrifuged at 100,000 g_av_ for 1 h at 4°C. The supernatant was discarded and the pellet containing the total membrane fraction was resuspended in 200-400 µL of 250 mM sucrose using a 1 mL-syringe and a 25G needle. 1 μL was used for the Bicinchoninic acid (BCA) assay (previously described) to determine protein concentration. The volume of the samples was adjusted with 250 mM sucrose to obtain a final protein concentration of approximately 25 µg/µL. The fractions were aliquoted in cryotubes, flash frozen in liquid nitrogen and stored at −80°C until use.

### 2.5 Thermal shift assay (TSA)

**FIGURE 1:**
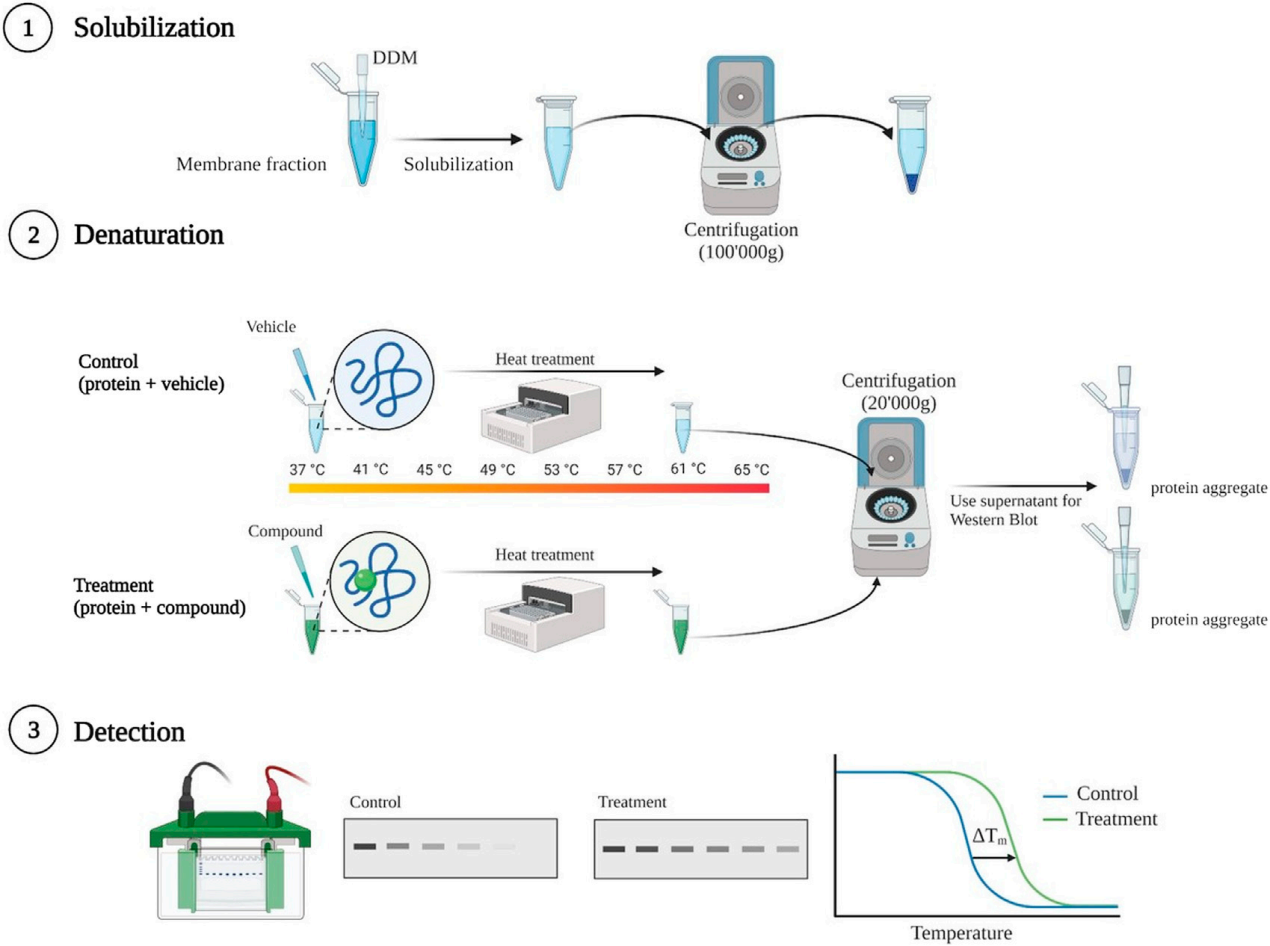
Schematic illustration of thermal shift assay procedure. Membranes harboring human OCT2 were solubilized using 1% Dodecyl-β-D-maltoside (DDM). Non-solubilized membranes were removed by ultracentrifugation. Thereafter, supernatant was incubated for 1 h at 4°C with either with the ligand at a final concentration of 1 mM, or with an equal volume of vehicle. After incubation equal volumes of samples were transferred into standard pcr tubes and heated at different temperatures in a thermocycler. Subsequent centrifugation removed the aggregates of denatured protein. The supernatant was prepared for SDS gel electrophoresis followed by Western blotting and band intensity quantification for melting curves generation. Created with BioRender.com.

Crude membranes were diluted to a final concentration of 1 μg/μL with 1% (w/v) n-Dodecyl-β-D-Maltopyranoside (DDM) (ThermoFisher Scientific, Waltham, MA, United States) dissolved in 150 mM NaCl, 20 mM Tris adjusted to pH 7.2 with HCl, and solubilized for 3 h in rotation at 4°C. Then, the sample was spun down at 100,000 g_av_ for 30 min at 4°C to clear the non-solubilized fraction. The supernatant was divided into two aliquots, which were incubated for 1 h at 4°C either with the ligand or with an equal volume of the vehicle. Then, each aliquot was distributed into thirteen 200 µL thin-wall tubes (standard PCR reaction tubes), each containing 30 μL (approximately 30 μg of proteins). Samples were kept at room temperature (non-heated control) or heated at different temperatures using a thermocycler with thermal gradient option. After 15 minute-incubation, samples were cooled down at room temperature and then centrifuged at 20,000 g_av_ to pellet protein aggregates. The supernatants were further denatured in Laemmli buffer containing 4 mM DTT for 5 min at 95°C for SDS gel electrophoresis and Western blot analysis. A schematic illustration of thermal shift assay procedure is shown in Figure 1.

### 2.6 SDS gel electrophoresis and western blotting

Samples were loaded onto an 8% polyacrylamide gel and resolved at a constant voltage of 100 V and then electroblotted either onto nitrocellulose membranes (GE Healthcare, Piscataway, NJ, United States) or polyvinylidene difluoride (PVDF) membranes (GE Healthcare, Piscataway, NJ, United States) for 1 h at 100 V. Subsequently, the membranes were blocked for 30 min in 5% (w/v) nonfat milk dissolved in PBS supplemented with 0.1% (w/v) Tween-20 (PBS-T), and then incubated overnight at 4°C with an antibody against the human OCT2 (640438, R&D Sytems, Minneapolis, MN, United States) 1:1000 diluted in 5% (w/v) nonfat milk. After three washings with PBS-T, membranes were probed for 1 h at room temperature with an ECL™ Anti-Mouse IgG horseradish peroxidase linked whole antibody (NA931, Sigma-Aldrich, St. Louis, MO, United States). Finally, membranes were exposed to the SuperSignal West Femto Maximum Sensitivity Substrate (ThermoFisher Scientific, Waltham, MA, United States) and the signal acquired in a Fusion FX7 instrument (Vilber Lourmat, Eberhardzell, Germany). Band intensity quantification was performed with the Image Lab Software version 6.0.1 (Bio-Rad, Hercules, CA, United States).

### 2.7 Protein modelling and docking

The AlphaFold model (Jumper et al., 2021; Varadi et al., 2022) of human OCT2 (UniProt O15244) was processed using the Protein Preparation Workflow of Maestro (Schrödinger Inc.) employing default settings for protonation, hydrogen bond optimization and restraint minimization. The processed OCT2 structure was embedded into a Dipalmitoylphosphatidylcholine (DPPC) model membrane, solvated with TIP3P water, neutralized in 150 mM NaCl and subjected to a 250 nanoseconds molecular dynamics simulation in Desmond (NVP mode, 310K, 1 bar, 2500 Frames). The workflow as well as the original AlphaFold model including the confidence scores of the prediction is shown in Supplementary Figure S1. Membrane embedded residues were identified with the PPM3 Web server (Lomize et al., 2022). The OCT2 protein conformations of the last 150 nanoseconds of the trajectory were clustered based on backbone RMSD (Supplementary Figure S2) and the cluster with the highest number of members was selected as representative OCT2 structure for subsequent studies. Docking of N-methylphenylpyridinium (MPP^+^) and select drugs to OCT2 was performed with Glide (Friesner et al., 2004; Halgren et al., 2004) (Schrödinger Inc.) by using the extra precision (XP) mode (Friesner et al., 2006) and a protein grid with a size of 36 Å positioned to include the extracellular part of the protein (Supplementary Figure S3). Binding free energies of the poses obtained including the contribution of different types of interactions (Coulomb, H-bond, van der Waals) were estimated with the MM-GBSA method (Genheden and Ryde, 2015) implemented in the Schrödinger Suite and with implicit membrane simulation and minimization of residues around 5 Å of the ligand. The optimized protein model of OCT2 as well as the docking poses have been deposited as pdb files for public access (DOI: 10.5281/zenodo.7692058).

### 2.8 Data analysis

The IC_50_ value calculation and statistical analyses were performed with GraphPad Prism (Version 9.4.1, GraphPad Software, San Diego, CA). For comparisons between two groups, Student’s *t*-test was applied.

## 3 Results

### 3.1 Effect of varying substrates on OCT2-mediated MPP^+^ uptake

**FIGURE 2:**
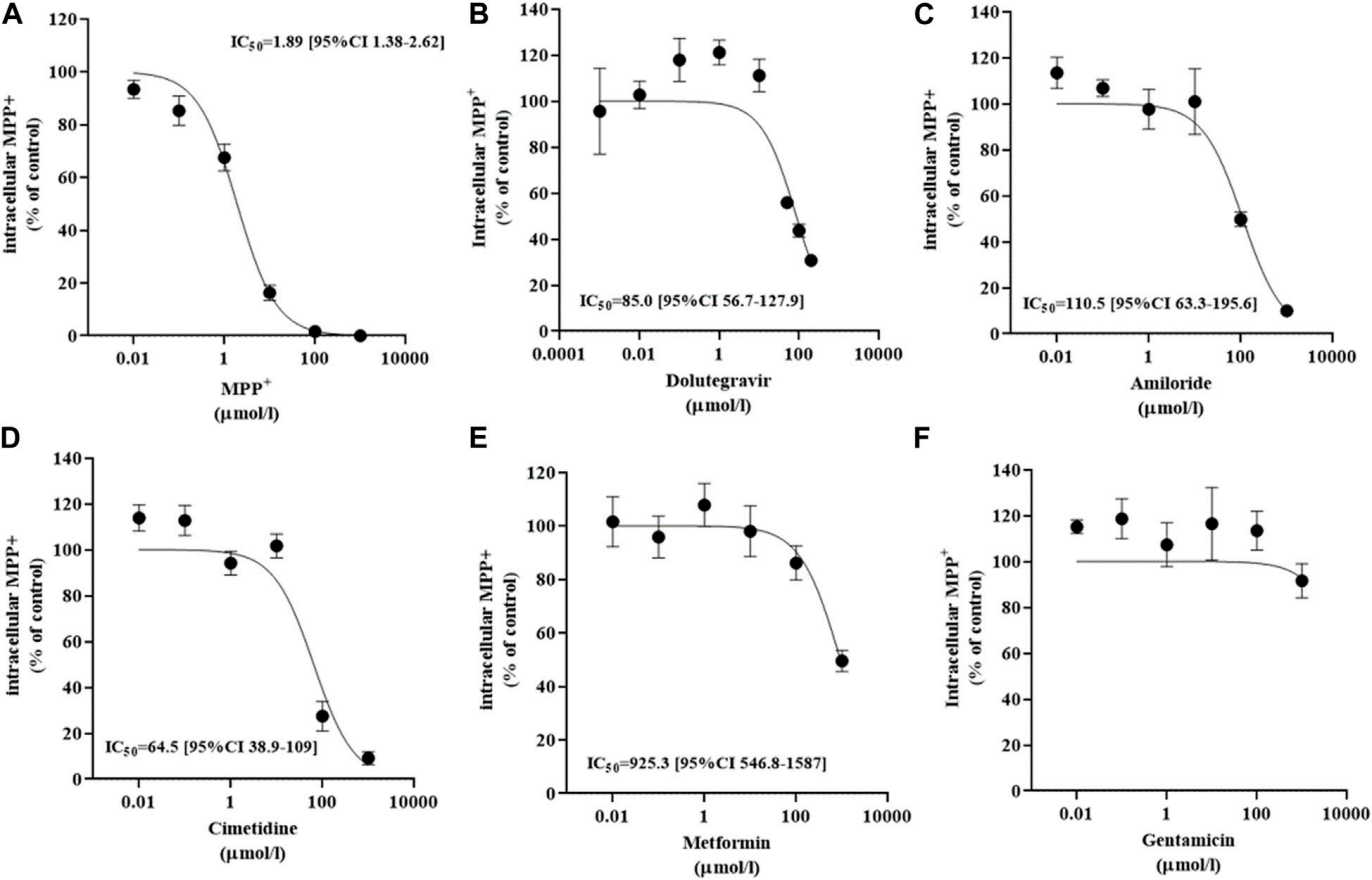
Inhibition of MPP^+^ uptake mediated by OCT2. Influx of MPP^+^ at an extracellular concentration of 0.5 µM in OCT2-HEK293 cells in the presence of increasing extracellular concentrations of MPP^+^ **(A)**, dolutegravir **(B)**, amiloride **(C)**, cimetidine **(D)**, metformin **(E)**, and gentamicin **(F)**. The uptake value measured in OCT2-HEK293 cells in the presence of the highest extracellular concentration of non-labelled MPP^+^ was subtracted to define the OCT2-specific transport. Data are expressed as percentage of the control and represent the mean ± SD from at least two independent experiments performed in duplicate. The IC_50_ values were computed from a variable slope model of the log (inhibitor) vs response curve.

Transport of 0.5 µM MPP^+^ was performed over 10 s, when the uptake in OCT2-HEK293 is unidirectional (Hormann et al., 2020). To define the OCT2-specific transport, (^3^H) MPP^+^ uptake rates in the presence of a saturating extracellular concentration of non-labeled MPP^+^ (1 mM) (Figure 2A) was used as background signal. A dose-dependent inhibition was observed when MPP^+^ uptake was assessed in the presence of increasing extracellular concentrations of dolutegravir (Figure 2B), amiloride (Figure 2C) or cimetidine (Figure 2D), with comparable computed IC_50_ values. Metformin (Figure 2E) and gentamicin (Figure 2F) did not show dose-dependent inhibition of MPP^+^ uptake in the concentration range used, although a partial inhibition was observed at the highest metformin extracellular concentration tested (1 mM). In a separate set of experiments, a partial inhibition of OCT2-mediated MPP^+^ uptake was observed in the presence of gentamicin at the extracellular concentration of 10 mM (not shown).

### 3.2 Prediction of ligand binding sites

**FIGURE 3:**
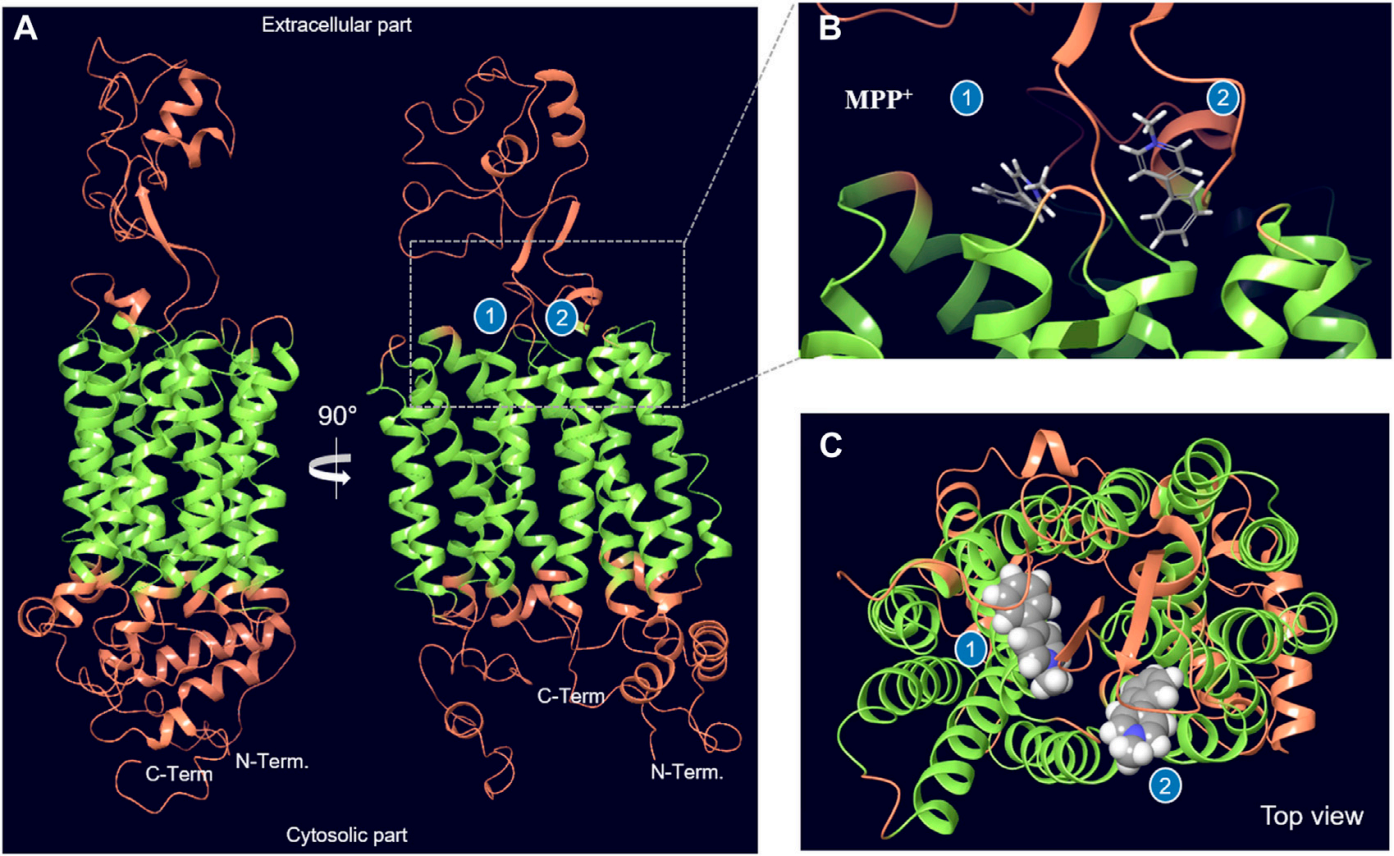
Optimized structural model of OCT2. Two distinct binding sites at the interface of the extracellular part and the transmembrane helices (in green) can be identified by docking of the model substate MPP+. The region of OCT2 that was used for docking is shown in Supplementary Figure S3. The estimated binding free energy for MPP+ in binding site 1 is –54.1 kcal/mol and for binding site 2 is –60.4 kcal/mol.

**FIGURE 4:**
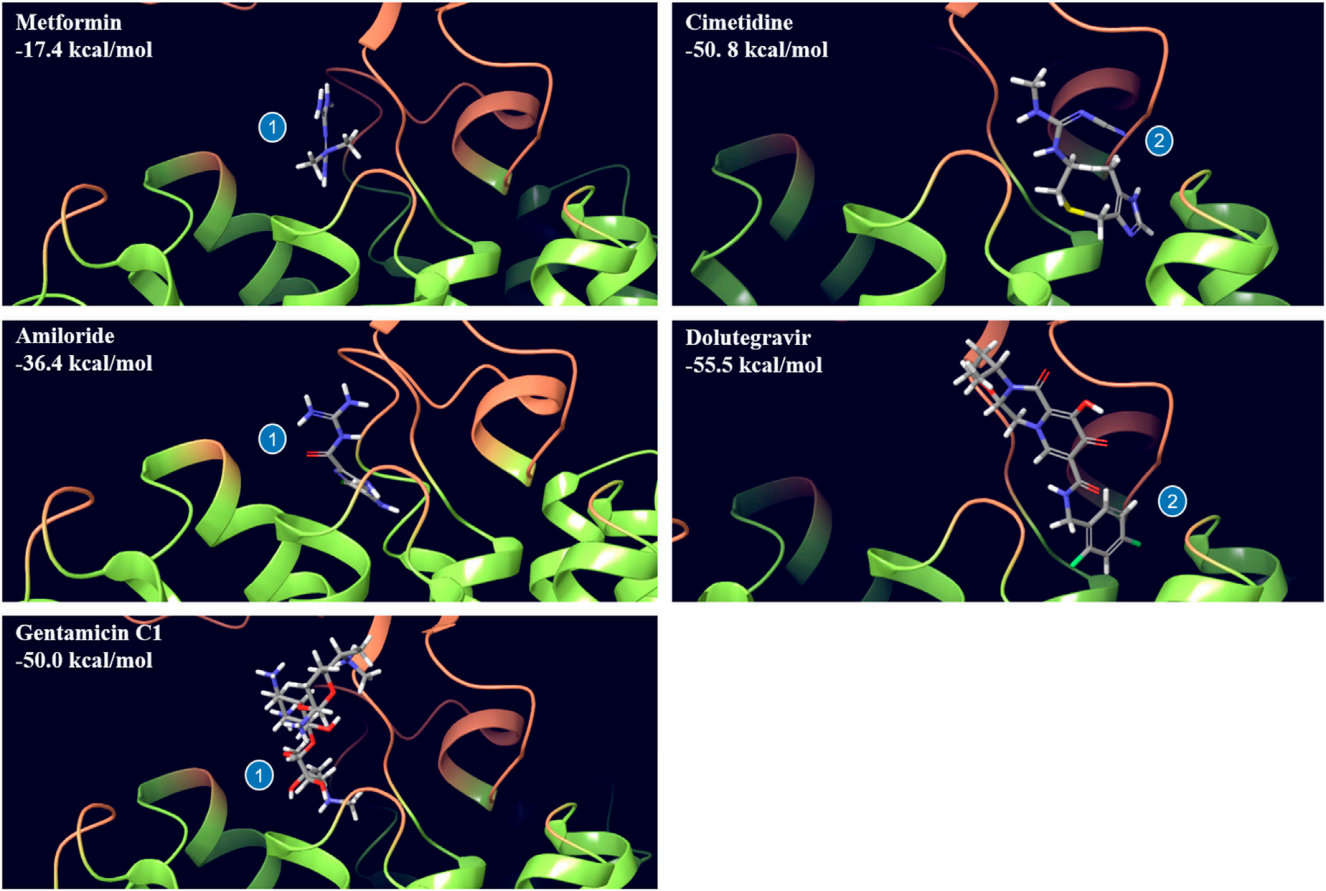
Binding pose of selected drugs to distinct binding sites of OCT2 as obtained from docking. Metformin, amiloride and gentamycin C1 bind to site 1, whereas cimetidine and dolutegravir bind to site 2. The estimated binding free energy of each pose is indicated in each panel.

The Alphafold structure of human OCT2 (UniProt O15244) after molecular dynamics based optimization is illustrated in Figure 3. Molecular docking of MPP^+^ molecules was conducted to the outer part of OCT2 cleft to pinpoint energetically favorable binding domains. The blind simulation posed two MPP^+^ molecules with no apparent steric hindrance, suggestive of two non-overlapping binding sites (Figure 3 and Supplementary Figure S4). Molecular docking of the ligands used in the *cis*-inhibition assay posed metformin, amiloride and gentamicin on binding site 1, whereas cimetidine and dolutegravir on binding site 2 (Figure 4). Details on the type of interaction stabilizing the binding of each ligand can be found in Supplementary Figures S5, S6. Although the OCT2 structure is only an optimized prediction, the molecular docking was in line with the *cis*-inhibition results, indicating that the substrates selected might bind in distinct portions of the binding pocket and that the choice of the model substrate and the concentration thereof is critical for the detection and quantification of the subsequent interaction with the inhibitor.

### 3.3 Ligand-induced thermal stability of OCT2

**FIGURE 5:**
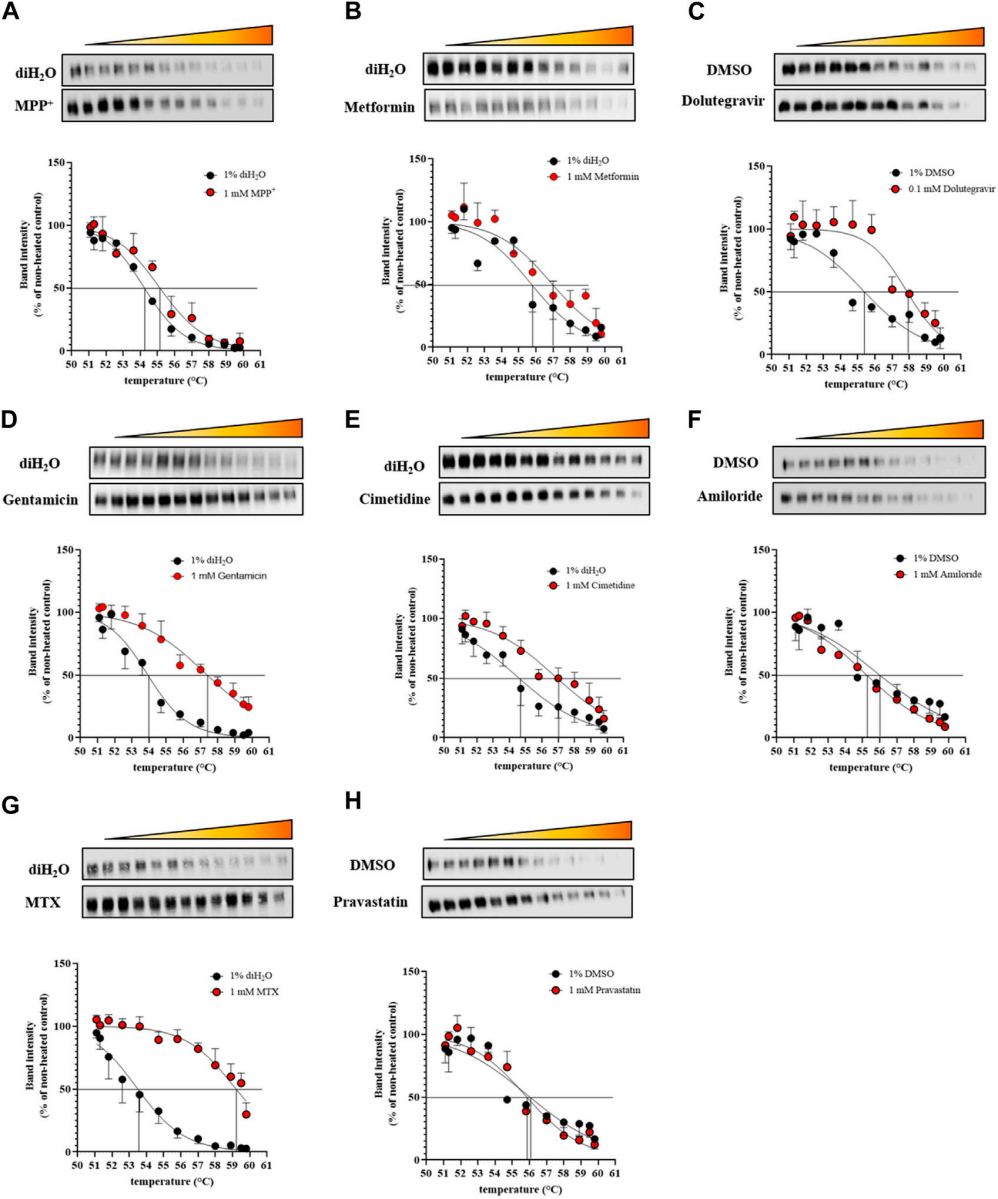
Melting curves of OCT2. DDM-solubilized membranes containing the human OCT2 were exposed to the indicated ligands at the concentration of 1 mM. Representative Western blot and relative quantification of the OCT2 remaining in solution after heating and centrifugation. Melting curves were generated from densitometric values at 12 different temperatures normalized to that of the non-heated control and fitted to a dose–normalized response equation with a variable Hill slope.

**FIGURE 6:**
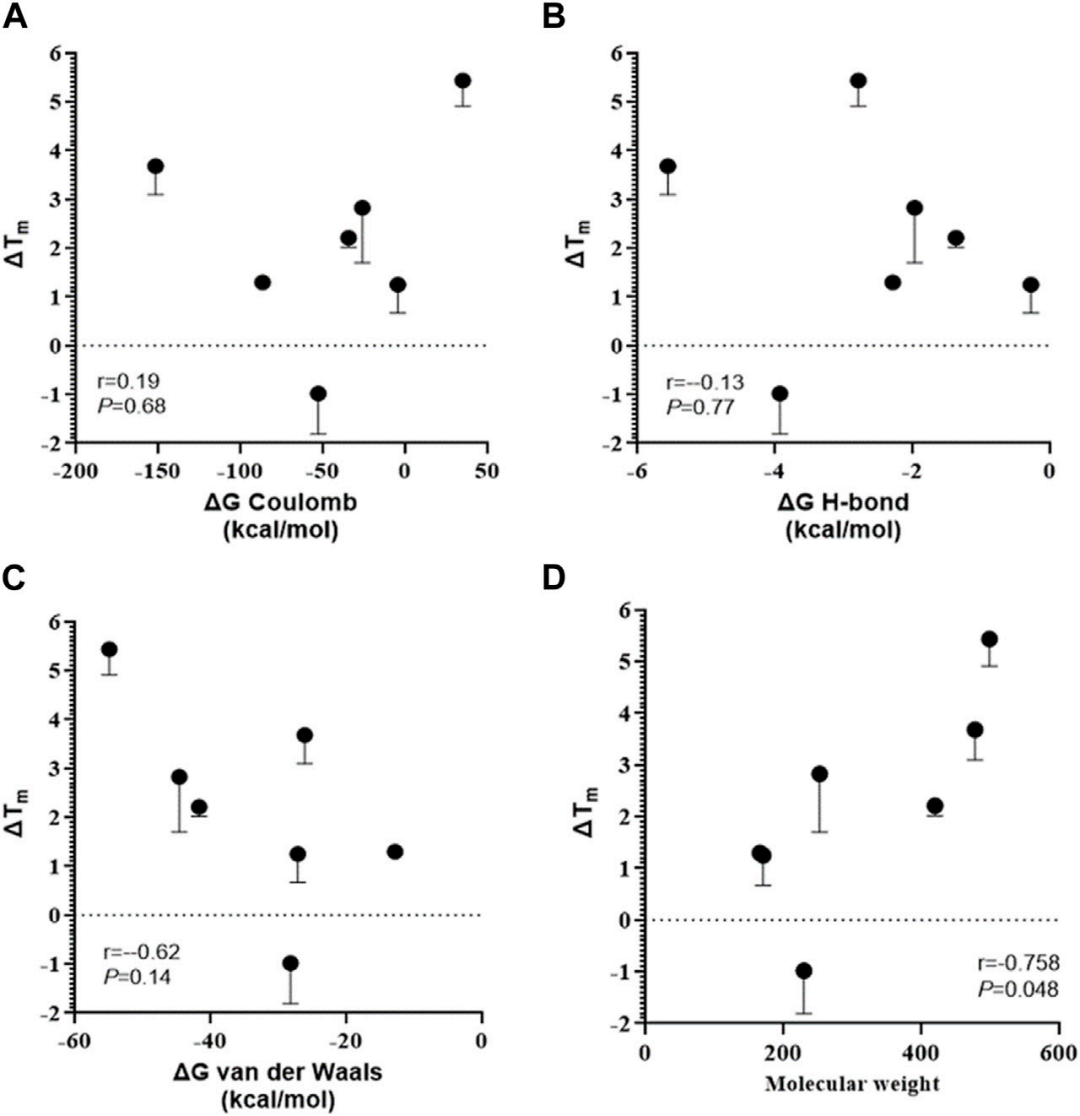
Correlation between thermal shift and ligand chemical structure. Thermal shift was calculated as the difference between the melting temperatures in the presence and absence of a ligand (ΔT_m_). Pearson’s Rank-Order analyses between the ΔT_m_ and the predicted energy of coulomb **(A)**, hydrogen-bond **(B)**, van der Waals interactions **(C)** or the molecular weight **(D)**.

**FIGURE 7:**
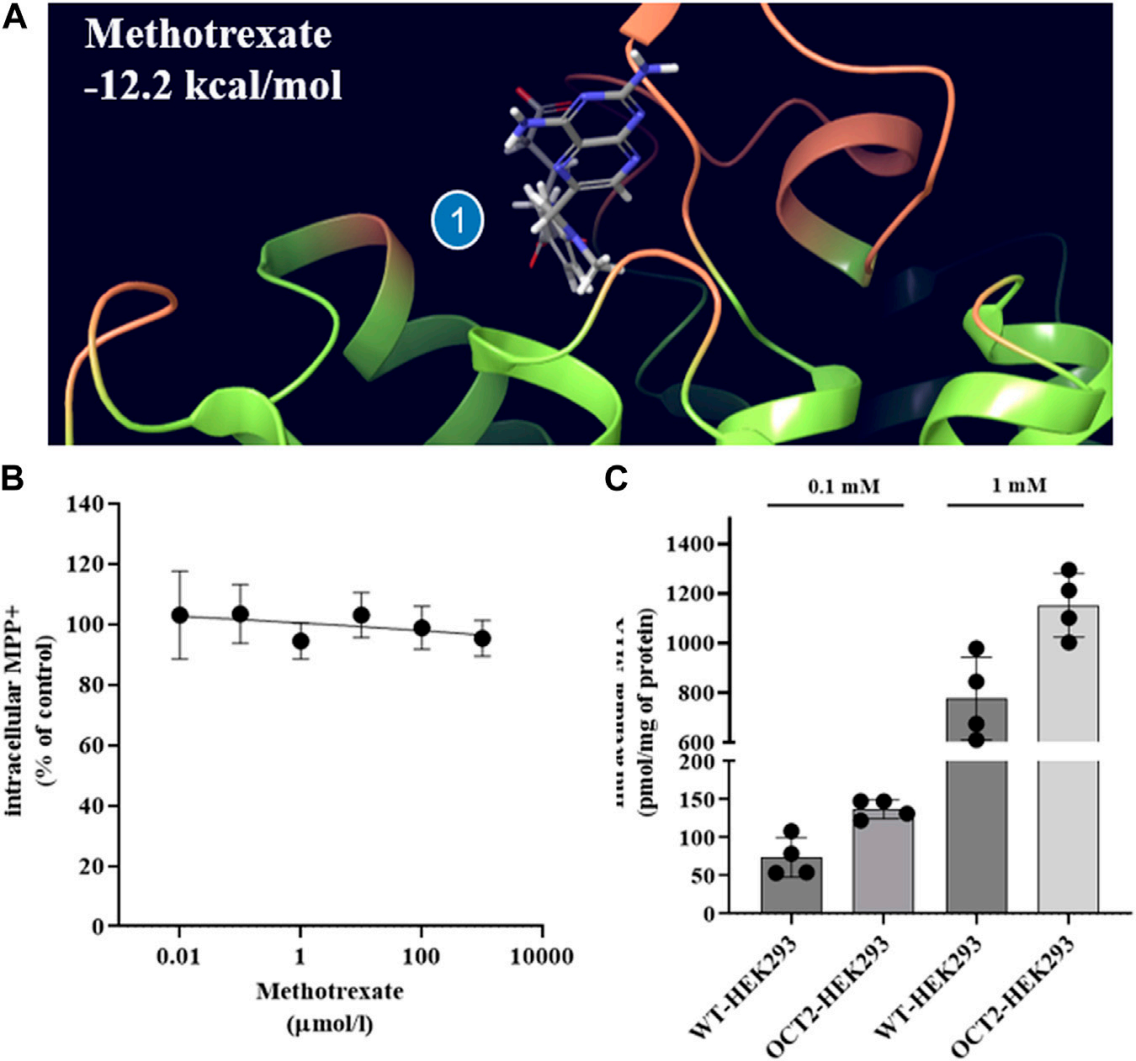
OCT2-MTX interaction in intact cells. Binding pose and estimated binding free energy of MTX to predicted binding site one of OCT2 **(A)**. Ten-second uptake of MPP^+^ at the extracellular concentration of 0.5 µM in OCT2-HEK293 cells in the presence of increasing extracellular concentrations of methotrexate (MTX). The uptake value measured in OCT2-HEK293 cells in the presence of non-labelled MPP^+^ at the extracellular concentration of 1 mM was subtracted to define the OCT2-specific transport. Data are expressed as percentage of the uptake value in vehicle-treated cells (control) and represent the mean ± SD from two independent experiments performed in duplicate **(B)**. One-minute uptake of MTX at the extracellular concentration of 1 mM in WT- and OCT2-HEK293 cells. *p*-value was calculated from unpaired *t*-test comparison of the mean ± SD from two independent experiments performed in duplicate **(C)**.

The same set of ligands was employed to investigate the effect of binding on the thermal stability of OCT2. Additionally, MTX and pravastatin were included as negative control. DDM-solubilized membranes containing the human OCT2 were exposed to different ligands at the highest concentration used in the *cis*-inhibition assay to maximize the OCT2 bound:unbound ratio, and then denatured at different temperature to obtain the melting curve of OCT2. The melting curve was used to compute the melting temperature (T_m_), at which half of the OCT2 molecules are denatured. The T_m_ of OCT2 in the absence of ligand was 54.4°C (95% CI 54.1-54.8). The incubation with 1% (v/v) DMSO increased, albeit not significantly, OCT2 thermal stability (55.6°C, 95% CI 55.1-56.2). The *cis*-inhibition results indicate that at the highest concentration of MPP^+^, dolutegravir, cimetidine or amiloride, all OCT2 molecules are occupied (Figure 2). However, the magnitude of the thermal shift (ΔT_m_) with respect to the vehicle control was obviously different amongst these ligands. Dolutegravir (Figure 5C) and cimetidine (Figure 5E) induced a larger shift of OCT2 melting curve than MPP^+^ (Figure 5A). Surprisingly, amiloride did not increase OCT2 thermal stability (Figure 5F). Metformin (Figure 5B) and gentamicin (Figure 5D), which showed partial and no inhibition of MPP^+^ uptake, respectively, did induce thermal stabilization of OCT2. Ligand specificity was assessed using MTX and pravastatin, two negatively charged molecules expected not to bind to OCT2. While pravastatin did not affect OCT2 thermal stability, MTX incubation induced an obvious shift of OCT2 melting curve with an apparent T_m_ of 59.2°C, representing the largest T_m_ shift among the ligand tested. T_m_ values for all the ligand tested are summarized in Table 1. Taken together, these results indicate no correlation between ΔT_m_ and IC_50_ values. Because ligand-thermal stability depends primarily on the enthalpic and entropic contributions to the binding free energy (Niesen et al., 2007), we assessed the possible correlation between the magnitude of the ΔT_m_ and the theoretical binding events calculated from the molecular docking. In Figure 6 it can be seen that the ΔT_m_ did not correlate with typical enthalpic factors such as Coulomb (Figure 6A), hydrogen (Figure 6B), and van der Waals (Figure 6C) interactions. Conversely, a positive correlation was found between the ΔT_m_ and the molecular weight of the ligand (Figure 6D), hence its chemical complexity, which typically positively correlates with the entropy of a system. Interaction with non-cationic substrates has been previously reported for human OCT1 and OCT2 (Kimura et al., 2002; Boxberger et al., 2018; Redeker et al., 2022); yet the obvious thermal stabilization upon incubation with MTX, a divalent anion, was unexpected, hence validated in intact cells by *cis*-inhibition of MPP^+^ uptake and by direct measurement of (^3^H) MTX uptake. Docking predicted MTX to bind to site one of OCT2 model (Figure 7A and Supplementary Figure S7). Figure 7B shows that OCT2-mediated uptake of MPP^+^ was not affected by the co-incubation with non-labeled MTX, irrespective of the concentration used. Nonetheless, the intracellular level of MTX was higher in the OCT2-HEK293 cells in comparison with that in WT-HEK293 cells after 1 minute-incubation with MTX at the extracellular concentration of 0.1 mM (137.4 vs. 73.7 pmol/mg of protein, *p* = 0.005) or 1 mM (1152.2 vs. 776.6 pmol/mg of protein, *p* = 0.01) (Figure 7C), indicating that MTX is a substrate of OCT2.

**TABLE 1:** Summary of OCT2 melting curves.

Ligand	Concentration	T_m_ (°C)	95% CI	ΔT (°C)	*p*-value
diH_2_O (*n* = 4)	1% (v/v)	54.2	54.0-54.5		-
MPP^+^ (*n* = 4)	1 mM	55.1	54.6-55.6	0.9	0.04
diH_2_O (*n* = 3)	1% (v/v)	55.7	55.2-56.3		-
Metformin (*n* = 3)	1 mM	57.0	56.3-57.7	1.3	0.007
diH_2_O (*n* = 4)	1% (v/v)	54.6	54.0-55.2		-
Cimetidine (*n* = 4)	1 mM	56.9	56.4-57.5	2.3	0.05
DMSO (*n* = 3)	1% (v/v)	55.4	54.8-55.9		-
Dolutegravir (*n* = 3)	0.1 mM	57.9	57.3-58.4	2.5	0.007
diH_2_O (*n* = 5)	1% (v/v)	53.9	53.6-54.3		-
Gentamicin (*n* = 5)	1 mM	57.4	56.9-57.9	3.5	0.003
DMSO (*n* = 3)	1% (v/v)	56.0	55.3-56.7		-
Amiloride (*n* = 3)	1 mM	55.2	54.8-55.7	−0.8	0.35
diH_2_O (*n* = 4)	1% (v/v)	53.5	53.0-54.0		-
Methotrexate (*n* = 4)	1 mM	59.2	58.8-59.8	5.7	0.002
DMSO (*n* = 3)	1% (v/v)	56.0	55.4-56.7		-
Pravastatin (*n* = 3)	1 mM	55.8	55.3-56.4	−0.2	0.17

Apparent melting temperature (*T*_m_) values were calculated from OCT2 densitometric values at 12 different temperatures normalized to that of the non-heated control and fitted to a dose–normalized response equation with a variable Hill slope. Paired *t*-test was used for comparison with the T_m_ values obtained from the matched control samples treated with 1% diH_2_O or 1% DMSO.

## 4 Discussion

Protein thermal stabilization induced by a ligand depends on the contributions of enthalpy (ΔH°) and entropy (ΔS°) of binding (Niesen et al., 2007). The binding enthalpy describes the strength of the interactions of the ligand with the protein (e.g., van der Waals, hydrogen bonds, etc.) relative to those existing with the solvent. The entropy change is constituted by solvation entropy and conformational entropy. Upon binding, desolvation occurs, water molecules that interact with the ligand and/or with the binding site in the unbound state are displaced upon binding of the ligand and a gain in solvent entropy is measured. At the same time, the ligand and certain groups in the protein lose conformational freedom, resulting in a negative change in conformation entropy (Leavitt and Freire, 2001; Williams et al., 2004). Typically, an increase in entropy is accompanied by a decrease in enthalpy (or *vice versa*) (enthalpy/entropy compensation principle) (Williams et al., 2004). When ΔS° >> ΔH°, binding of the ligand to the target protein is entropically driven as a result of water molecule displacement and it is typically characterized by greater thermal stability (Niesen et al., 2007), thus a larger thermal shift (ΔT_m_) in comparison with an enthalpically driven binding, where ΔH° >>ΔS°. Because more complex and bigger molecules can potentially displace more water molecules bound to the binding site than smaller ones, resulting in a higher entropy of the solvent (Sabirov and Shepelevich, 2021), it is not surprising that the ΔT_m_ induced by a relatively strong substrate such as MPP^+^ was narrower than that induced by gentamicin and MTX, which could be considered to be weaker substrates of OCT2, but have a greater mass and chemical complexity than MPP^+^. The positive correlation between the molecular mass of the ligand and OCT2 ΔT_m_ but not the number or type of (enthalpic) interactions, suggests that solvation plays an important role in tuning binding affinity for OCT2.

No correlation was found between the IC_50_ values computed from the *cis*-inhibition results and the magnitude of the ΔT_m_, indicating that the thermal stabilization induced by a ligand is not a reflection of the relative binding affinity. ΔH° and ΔS° are the two components of the standard free energy of the binding equilibrium (ΔG° = ΔH°—TΔS°), which dictates the binding affinity and can also be calculated from the dissociation constant K_D_ (ΔG° = RT ln K_D_). Therefore, many combinations of ∆H and ∆S values can, in principle, elicit the same binding affinity, hence K_D_ values provide limited information on the molecular mechanisms underlying the binding (Borea et al., 2000; Niesen et al., 2007). Conversely, the ΔT_m_ values offer information on the chemical characteristics of the binding irrespective of the strength. For example, amiloride, and dolutegravir have comparable IC_50_ values of MPP^+^ uptake (Figure 2) but dolutegravir-induced thermal stabilization of OCT2 was substantial; resulting in an obvious shift of the OCT2 melting curve, whereas amiloride did not increase OCT2 thermal stability at all, suggesting that the binding modality of these two drugs to OCT2 is rather different, despite the comparable inhibitory effect on MPP^+^ uptake. Considering IC_50_ values proxy for the K_D_ values, hence the calculated ΔG° (ΔG° = RT ln K_D_) of the binding equilibrium for amiloride (−23.5 kcal/mol) and dolutegravir (−24.2 kcal/mol) are also similar (Borea et al., 2000). We could speculate that the binding of OCT2 to dolutegravir is entropically driven, while that to amiloride is enthalpically driven. The TSA might provide qualitative information on the entropic and enthalpic contribution to the binding to OCT2 that can be used as a further physicochemical parameter for pharmacophore modeling (Zolk et al., 2009; Belzer et al., 2013; Hacker et al., 2015).

Regulatory Agencies mandate each new molecular entity (NME) to be tested *in vitro* as inhibitor of all clinically relevant transporters, including OCT2 (European Medicines Agency (EMA), 2015; United States Food and Drug Administration (FDA), 2020). The simplest experimental approach is measuring the uptake of one prototypical substrate in the presence of increasing concentrations of the NME. The ratio between the NME *in vivo* maximum unbound plasma concentration and the *in vitro* half-maximal inhibitory concentration value (IC_50_) defines DDI potential and the need of initiating a clinical DDI study. This approach has shown low sensitivity in the prediction of OCT2 inhibition, arguably because several ligands can inhibit OCT2-mediated transport in a non-competitive or mixed fashion (Harper and Wright, 2013) and because the ligand spatial interaction with OCT2 as well as the strength thereof is conditional to the model substrate (Keller et al., 2019; Hormann et al., 2020; Sutter et al., 2021). Our data agree with the current model of OCT2 binding and support the validity of the proposal of adding at least one compound (e.g., metformin) to MPP^+^ as a complementary model substrate to increase the sensitivity of the DDI *in vitro* screening (Koepsell, 2021). The uptake of MPP^+^ at the extracellular concentration of 0.5 µM was completely inhibited and in a dose-dependent manner, by dolutegravir, cimetidine and amiloride, whereas metformin and gentamicin induced partial and no inhibition, respectively. IC_50_ values were comparable to other previously published using MPP^+^ as a model substrate (Koepsell, 2021) (12, 39). Indeed, molecular modeling and docking analysis revealed two binding sites for MPP^+^, site 1 is shared with metformin, gentamicin and amiloride, site 2 with cimetidine and dolutegravir. Arguably, at the extracellular concentration of 0.5 µM, MPP^+^ saturates site 2, competing with dolutegravir and cimetidine, and partially binds to site 1, where metformin and gentamicin might preferentially bind. In contrast with the docking results, it is possible that amiloride binds to both binding sites with similar likelihood.

In conclusion, when looking at the rather trivial effect on OCT2 thermal stability induced by relatively strong substrates such as MPP^+^, metformin and amiloride, TSA does not seem to represent a viable approach for the identification of small, chemically simple ligands, even those characterized by high affinity. Conversely, TSA might be useful in pinpointing substrates with greater chemical complexity, which are probably characterized by higher solvation costs (high entropy), hence larger ΔT_m_ (Bronowska, 2011; Harper and Wright, 2013; Keller et al., 2019; Hormann et al., 2020; Sutter et al., 2021), as observed for dolutegravir, gentamicin and MTX. This works offers an unprecedent perspective on OCT ligand characterization, whereby the energy required to disrupt water-protein interactions is likely to be a rather important element in ligand binding. We speculate that the hydrophobic interaction domain that characterizes the modeled OCT pharmacophore (Zolk et al., 2009; Belzer et al., 2013) facilitates the disruption of water-water, and water-protein hydrogen bonds, thereby reducing the entropic costs for binding.

## Data Availability

The original contributions presented in the study are included in the article/Supplementary Material, further inquiries can be directed to the corresponding author.
