# Supplementary material for: Characterization of ligand-induced thermal stability of the human organic cation transporter 2 (OCT2)
Source: Front Pharmacol. 2023 Mar 16;14:1154213. doi: 10.3389/fphar.2023.1154213 (PMC10061065; doi:10.3389/fphar.2023.1154213)
Supplement: Supplementary file 1 [file DataSheet1.docx]

Supplementary Material to

Characterization of ligand-induced thermal stability of the human organic cation transporter 2 (OCT2)

Max Maane, Fangrui Xiu, Peter Bellstedt, Gerd A. Kullak-Ublick, and Michele Visentin


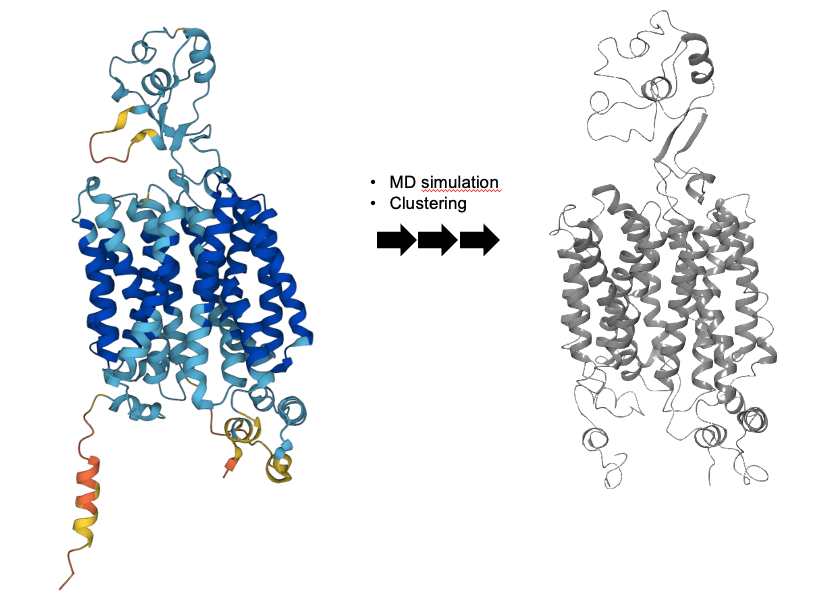


- MD simulation
- Clustering

**Supplementary Figure 1. Processing of the OCT2 Alphafold model.** The predicted structure of OCT2 (Uniprot O15244) on the left contained low (yellow) to very low (orange) per-residue confidence scores in both the extracellular as well as the intracellular part of the protein, wheras the transmembrane region were predicted as confident (light blue) or very confident (dark blue). After subjecting the alphafold model to a 250 ns molecular dynamics (MD) simulation and clustering of the protein conformers we observed partial reorganisation of structural elements, especially of the intracellular helix which was predicted only with very low confidence in the original model. As the RMSD during the MD simulation stabilized after the first 100 ns, we considered the processed model of OCT2 (depicted in grey) more realistic and used this model for docking. The final model has been deposited for public access (DOI: 10.5281/zenodo.7692058) .

**
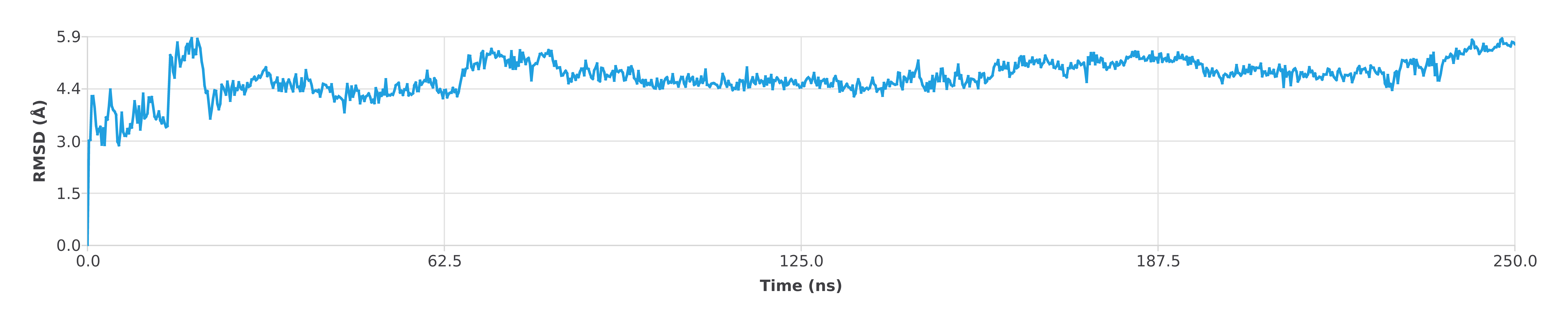
**

**Supplementary Figure 2. All Atom Root Mean Square Deviation of the OCT2 Alphafold model during molecular dynamics simulation.** The last 150 ns (red box) have been used for clustering to obtain the final model.


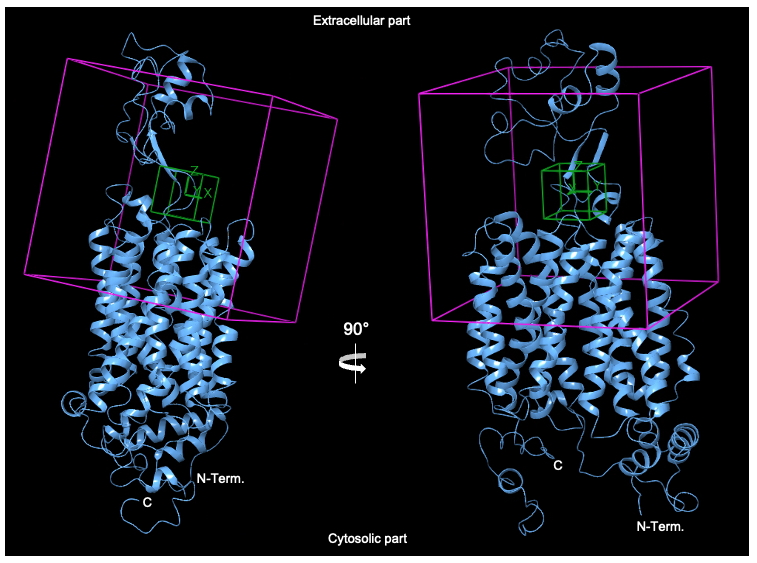


**Supplementary Figure 3. Part of the OCT2 model used of Docking.** The cubic docking grid (purple box) was positioned to include the extracellular part of the transporter as well as the interface to the membrane and had a size of 36 Å, which is the maximum technical size of a grid in the software used.


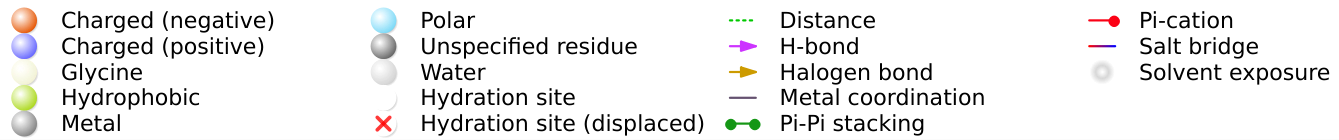


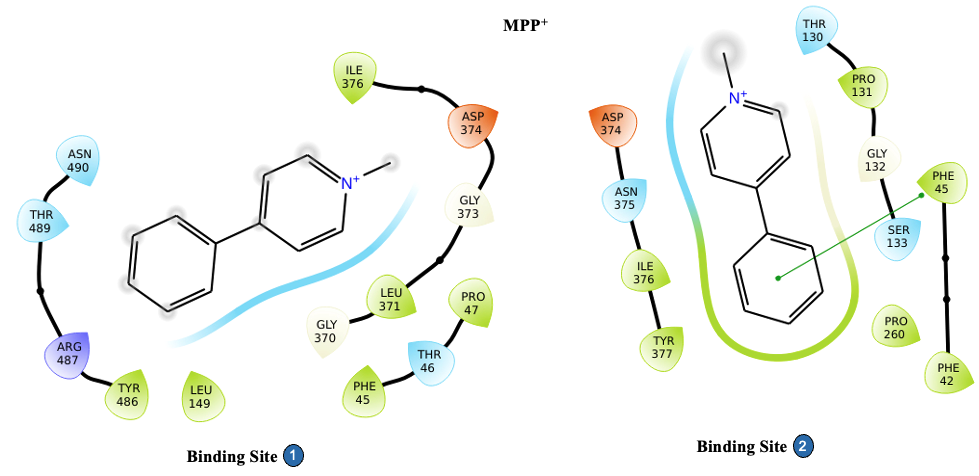


**Supplementary Figure 4. Interactions of N-methylphenylpyridinium (MPP^+^) as obtained from docking to the extracelluar part of the OCT2 model.**


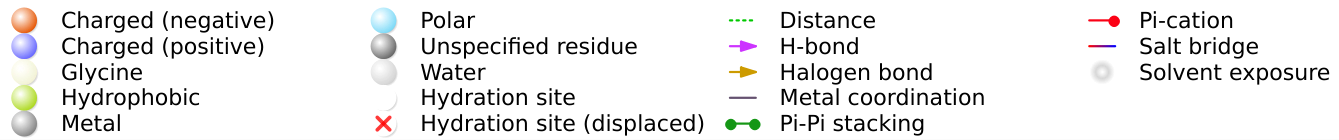

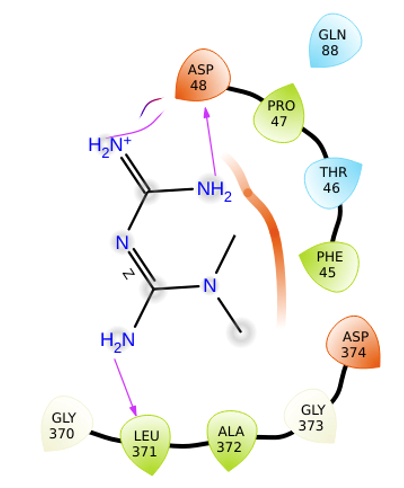

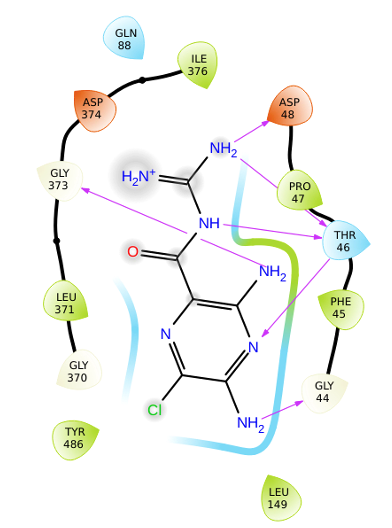

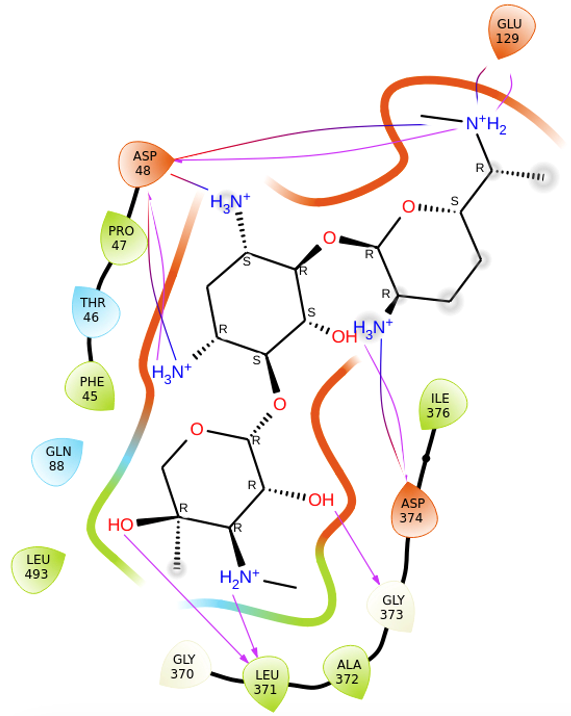


Metformin Amiloride Gentamicin C1

**Supplementary Figure 5. Interactions of Metformin, Amiloride and Gentamicin C1 as obtained from docking to the extracelluar part of the OCT2 model.**


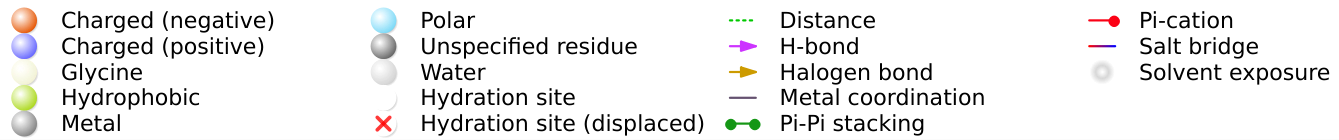

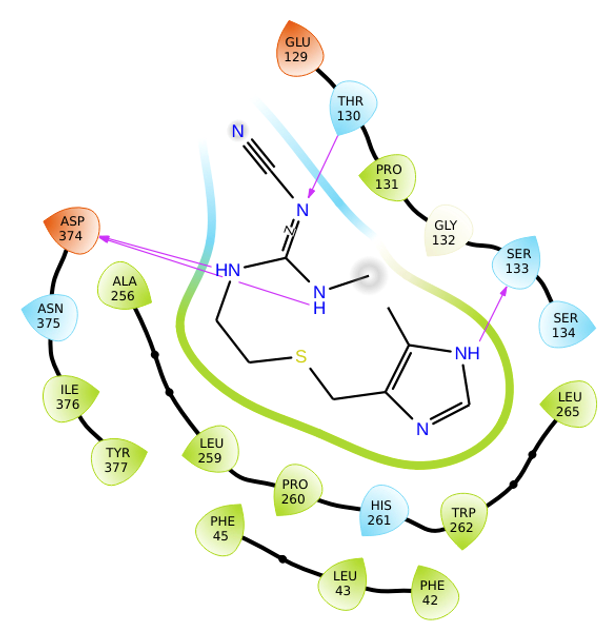

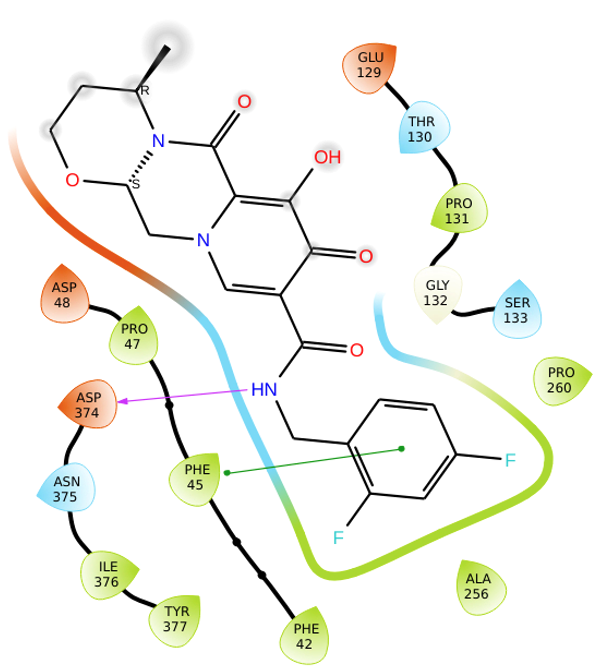


Cimetidine Dolutegravir

**Supplementary Figure 6. Interactions of Cimetidine and Dolutegravir with the OCT2 model as obtained from docking to the extracelluar part.**


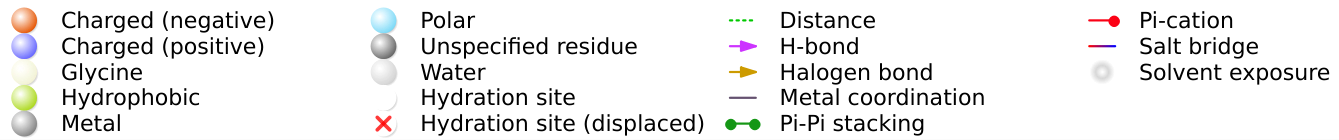


**
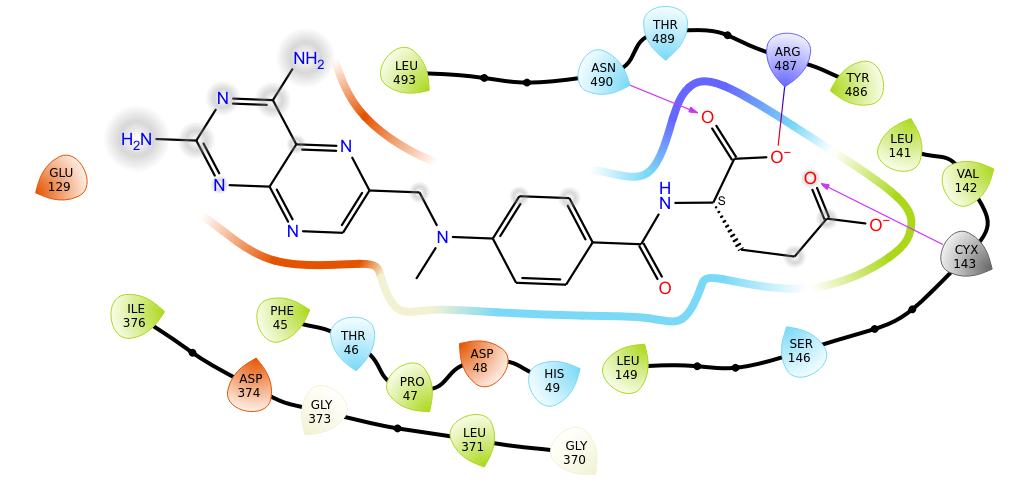
**

**Supplementary Figure 7. Interactions of Methotrexate with the OCT2 model as obtained from docking to the extracelluar part.**
